# Humin as an External Electron Mediator for Microbial Pentachlorophenol Dechlorination: Exploration of Redox Active Structures Influenced by Isolation Methods

**DOI:** 10.3390/ijerph15122753

**Published:** 2018-12-05

**Authors:** Duyen Minh Pham, Arata Katayama

**Affiliations:** 1 Department of Civil Engineering, Graduate School of Engineering, Nagoya University, Nagoya 464-8603, Japan; pham.duyen.minh@imass.nagoya-u.ac.jp; 2 Institute of Materials and Systems for Sustainability, Nagoya University, Nagoya 464-8603, Japan

**Keywords:** humin, PCP dechlorination, external electron mediator, redox-active function, quinone structure, peptidoglycan, aromaticity

## Abstract

Humin (HM) has been reported to function as an external electron mediator (EEM) in various microbial reducing reactions. In this study, the effect of isolation methods on EEM functionality and the chemical/electrochemical structures of HM were examined based on the correlation between dechlorination rates in the anaerobic HM-dependent pentachlorophenol (PCP)-dechlorinating consortium and the chemical/electrochemical structures of HM. A lack of PCP dechlorination activity suggested no EEM function in the HM samples prepared as a soluble fraction in dimethyl sulfoxide and sulfuric acid (which did not contain any electric capacitance). Other HM samples exhibited EEM functionality as shown by the dechlorination activity ranging from 0.55 to 3.48 (µmol Cl^−^) L^−1^d^−1^. The comparison of dechlorination activity with chemical structural characteristics suggested that HM with EEM functionalities had predominantly aliphatic and carbohydrate carbons with the partial structures C=O, O=C–N, and O=C–O. EEM functionality positively correlated with the proportion of O=C–N and O=C–O, suggesting an association between peptidoglycan structure and EEM functionality. The lack of detection of a quinone structure in one HM sample with EEM functionality and a negative correlation with aromatic or C=C carbon suggested that the mechanism containing quinone structures is a minor component for the functionality of EEM.

## 1. Introduction

The function of humic substances (HSs) as external electron mediators (EEM) in soil and sediment was found to reduce iron during microbial respiration by Lovely et al. [1]. This redox-active property of HSs has been studied extensively during the past two decades, but all of the studies focused on soluble fractions of HSs [2,3,4,5]. The soluble fractions of HSs are humic acid and fulvic acid, or quinoid analogues. Roden et al. [6] reported that solid-phase HSs have redox-active functions during enhanced iron-reducing microbial activity. However, humin (HM) has been considered as an inert fraction, despite the fact that HM is a major non-soluble fraction of HSs in soil and sediment [7]. For the first time, we found the redox-active function of solid-phase HM during the reductive dechlorination of pentachlorophenol (PCP) [8]. Since then, the function of HM as EEM has been explored, including that in the debromination of tetrabromobisphenol A [9] and iron and nitrate reduction [10]. This electroactive function of HM has been successfully further applied to a bioelectrochemical remediation system for the dechlorination of PCP [11] and for nitrate removal by denitrification [12]. These results suggest that HM is a useful EEM for bioelectrochemical remediation. HM is recalcitrant in the environment [13] and possesses the characteristics preferable for a bioelectrochemical remediation system. The loss from the bioremediations site used would be minimal because of the insoluble property of HM. The function of HM has been reported to be stable against the treatments with acid/alkali, by boiling/freezing, and oxidation/reduction [8].

While the applicability of HM as the EEM to bioremediation has been explored, the chemical structure of HM that is responsible for the bioelectroactive function has not been fully elucidated. The spectroscopic characterization of HM from different soils containing various clay types suggests that the quinoid structure is common in the organic fraction of HM as a redox active moiety in HM [8,14]. However, the different activities depending on HM have not been explained, likely due to the many factors affecting the activity in the limited number of soils. This indicates the need for a comparative study using HM with different organic chemical structures, while the clay minerals remain the same. It has been known that HMs prepared by differing isolation methods exhibit different organic chemical compositions and structures. These differences are due to changes in solubility with the isolation solutions, as well as the structural modification by the reaction with the isolation solutions [15,16,17,18,19,20]. Various isolation solutions such as HF, NaOH, HCl, H_2_SO_4_, methylisobutyl ketone (MIBK), dimethylsulfoxide (DMSO), and bromoform have been used to remove fulvic and humic acids and inorganic clay minerals [10,16,18,19,20,21,22,23,24,25]. The electroactive function of HM has only been demonstrated using HM isolated with 0.1 N NaOH and 2% HF [8].

Thus, we have conducted this study to compare seven different HM isolation methods from the same soil, based on the relation between the HM function as EEM in an HM-dependent PCP-dechlorinating microbial consortium. The chemical structure and electrochemical characteristics of HM were elucidated by the analyses of elemental composition, cyclic voltammetry, using the spectra of Fourier transform infrared spectroscopy (FT-IR), electron spin resonance (ESR), nuclear magnetic resonance (NMR), and synchrotron-radiation-based X-ray photoelectron spectroscopy (XPS).

## 2. Materials and Methods

### 2.1. HM Isolation Methods

Kamajima paddy soil (collected in Aichi Prefecture, Japan) was first air-dried (in dark conditions), then ground (by ceramic mortar) and sieved (by a 1.0 mm sieving size), to remove roots and stones. HM was then isolated from the soil using six different methods, with some modifications as summarized in Table 1. Schematic diagrams of six methods are shown in the supplemental information (Figures S1–S4). In the isolation procedures, 100 g of the sieved dried soil was placed in a centrifugation bottle and treated with 150 mL of the different solutions or solvents. In each treatment, the bottle was shaken at 110 revolutions per minute for 24 h. The solution and the residue were separated by centrifugation at 8000× *g* for 15 min. Then, the solution was discarded by decantation, and the residue was treated further with new solution and/or solvent, (unless otherwise noted).

#### 2.1.1. Method 1 (HM-M1)

This HM was isolated by a method previously reported by our laboratory [8], with some modifications. Briefly, the sieved dried soil was washed twice with 0.1 N NaOH to remove humic acid, then four times with 2% HF to remove amorphous silicates. The residue was then washed twice with 0.1 N NaOH and twice with 2% HF to remove additional humic acid, fulvic acid, metals, and biogenic compounds (such as proteins). The remaining residue was then washed twice with 0.1 N NaOH, followed by repeated washing with ultrapure water until a neutral pH was obtained and freeze-dried. The freeze-dried sample was subjected to the following experiments notated as the HM sample of method 1 (HM-M1).

#### 2.1.2. Method 2 (HM-M2)

The sieved dried soil was first washed twice with a 1:1 benzene/methanol (*v*/*v*) solution, then four times with 0.1 N NaOH. Silicates in NaOH-treated residue were then removed with concentrated HCl/HF (1:9 *v*/*v*), twice. Finally, the HM (with high organic content) was washed two more times with NaOH, then neutralized by repeated washings with ultrapure water, and freeze-dried in the end.

#### 2.1.3. Method 3 (HM-M3)

The sieved dried soil was washed three times with 0.1 N NaOH for 24 h. The NaOH-treated residue was then washed twice with MIBK (acidified to pH 1 with concentrated HCl). In this step, fulvic acid and mineral materials dissolve in the aqueous phase. The MIBK-treated residue was then washed three additional times with NaOH and neutralized with repeated washings using ultrapure water and then freeze-dried.

#### 2.1.4. Method 4 (HM-M4)

The sieved dried soil was first treated twice with 0.1 M H_3_PO_4_ to remove carbonates and mineral cations in soil. The residue was washed four times with 0.1 M Na_4_P_2_O_7_ and then one time with 0.1 N NaOH. A mixture of CHBr_3_ and ethanol (density = 1.8 g/cm^3^) was used for further treatment of residue. At this step, free organic matter (light fraction) was floated above the solvent mixture and was recovered using filter paper (Advantec No.1, pore size of 6 µm, Toyo Roshi Kaisha Ltd., Tokyo, Japan). The recovered organic fraction was washed one more time with 0.1 N NaOH, then neutralized by repeated washings with ultrapure water, and finally freeze-dried as HM.

#### 2.1.5. Method 5 (HM-M5)

The sieved dried soil was washed twice with 0.1 N NaOH, six times with 0.1 N NaOH containing 30 g/L Na_2_SO_4_, and twice with Na_2_SO_4_ (30 g/L). The residue was then washed two more times with 0.1 N NaOH, neutralized by repeated washings with ultrapure water, and then freeze-dried.

#### 2.1.6. Method 6 (HM-M6 and HM-M7)

The sieved dried soil was washed twice with 0.1 N NaOH, and then six times with 0.1 N NaOH containing 6 M urea. The residue was then washed twice with the mixture of dimethylsulphoxide (DMSO) and concentrated H_2_SO_4_ (6 % *v*/*v*). In this step, after centrifugation of each washing, dark supernatant and solid residue were both collected separately. Solid residue (low organic carbon content) was washed twice with 0.1 N NaOH, then neutralized by repeated washing with ultrapure water, freeze-dried, and finally named as HM-M6. The dark-colored supernatant was adjusted in pH 9 with saturated NaOH solution, while the soluble fraction was precipitated as organic-carbon rich HM and collected. This organic-carbon rich HM was washed twice with 0.1 N NaOH, neutralized by repeated washings with ultrapure water, then finally freeze-dried, and named HM-M7 for characterization.

### 2.2. Influence of HM on the Microbial Reductive PCP Dichlorination

The EEM function of HM isolated by the six methods were examined using the anaerobic HM-dependent PCP-to-phenol dechlorinating consortium, as described in Zhang and Katayama [8]. Briefly, each serum bottle containing 20 mL of mineral medium and 5 g/L of HM was first autoclaved. Then, 10 mM (final composition) of formate as carbon, an energy source, 20 µM of PCP, 0.2 mL of ×100 vitamin, and 5% (*v*/*v*) of microbial source were added to the bottle. The HM culture was incubated stably at 30 °C under dark conditions and maintained through 5% serial transfers (*v*/*v*). Zhang and Katayama [8] had confirmed that PCP was dechlorinated only in the culture containing both HM (corresponding to HM-M1 in this study) and the microbial consortium.

To evaluate the effect of HM on the activity of PCP dechlorination, the concentrations of PCP and metabolites were determined after the appropriate incubation time, using a QP2010 gas chromatography-mass spectrometry (Shimadzu, Kyoto, Japan) equipped with a DB-5MS column (J&W Scientific Inc., Folsom, CA, USA) [8,14,30]. In this experiment, two negative controls were provided: the culture without the addition of HM and the culture without the inoculation of the consortium. The culture amended with HM-M1 as EEM served as the positive control because this positive activity of HM-M1 has been confirmed by Zhang and Katayama [8], Zhang et al. [11], and Zhang et al. [14]. HM was judged to have electron mediating functionality when it maintained the PCP dechlorination in the culture after at least three generations of subcultures [8,9].

### 2.3. Chemical and Spectroscopic Characterization

#### 2.3.1. CHN Ash Analysis

A Yanaco MT-5 CHN-corder (Yanaco New Science Inc., Kyoto, Japan) was used to determine the elemental composition of HM, which included carbon (C), hydrogen (H), nitrogen (N), oxygen (O), and ash content. Antipyrine was used as a standard. Ash content was calculated from the remaining weight of the HM sample after the measurement. The oxygen content was calculated by subtracting the percentages of C, H, N, and ash from 100%.

#### 2.3.2. Fourier Transforms Infrared (FT-IR) Spectroscopy

The FT-IR spectra of HM were obtained from a JASCO FT-IR-6100 spectrometer (JASCO, Tokyo, Japan). The scanned wavenumber range was 4500 to 500 cm^−1^ with a resolution of 4 cm^−1^ and eight accumulation modes. The spectrum of pure potassium bromide, KBr, was used as the background for correction of the HM sample. Regarding sample preparation, HM was mixed with KBr homogenously and then pressed to form a pellet according to the KBr pellet technique [31].

#### 2.3.3. Electron Spin Resonance (ESR)

A JES-FA200 ESR spectrometer (JEOL Co., Ltd., Tokyo, Japan) was used to measure the ESR spectra of HM. HMs were placed in quart glass tubes (JEOL Co. Ltd., Parts No. 422000281, inner diameter of 4 mm) until it filled up to 40 mm from the bottom. Each HM was examined at pH 3 (adjusted using 0.1 M HCl) and pH 11 (adjusted using 0.1 M NaOH) to evaluate the effect of the pH of the HM on the ESR signal intensity. The spectrometer was operated at room temperature with these measurement conditions: frequency 9440 MHz with modulation of the steady magnetic field being 100 kHz, time constant 0.03 s, and modulation width of 0.2 mT.

#### 2.3.4. Nuclear Magnetic Resonance (NMR)

An ECA-700 spectrometer (JEOL Co. Ltd., Tokyo, Japan) was used to analyze solid-state ^13^C CP/MAS NMR spectra of the HM samples. HM was packed in a Zirconia sample tube (JEOL No. 780239954) and installed in the sample holder of the spectrometer. The measurement conditions were set as follows: relaxation time 1 s, contact time 2 ms, resonance frequency 176 MHz, and sweeping speed 70.4 kHz. HM-M1 and HM-M2 were conducted with 80,000 scans, while 160,000 scans were applied from HM-M3 to HM-M7. Hexamethylbenzene (HMB) was used as a standard sample to ensure the condition of the spectrometer.

#### 2.3.5. X-ray Photoelectron Spectroscopic (XPS) Analysis

The sample preparation technique for HM characterized by synchrotron based-XPS had been developed successfully recently by Pham et al. [32]. Briefly, HM was first mixed thoroughly with copper powder (size 75 µm, purity 99.95%) at a HM:Cu ratio of 1:1 *v*/*v* in a ceramic mortar. A stainless-steel dice set with a pellet size of five mm in diameter and a pressure about 175 MPa was used to form the pellet sample. This pellet sample was then attached onto the sample plate (SUS 316L, Japanese Industrial Standards) using both a double-sided adhesive carbon tape and a screw nut on the surface. XPS measurement were carried out using a Beamline 7U (ultra-soft X-ray, 32–1000 eV) at the Aichi Synchrotron Radiation Center (Aichi, Japan). The X-ray energy (hν) of a wide scan was 900 eV, and of narrow C 1s scan was 650 eV. XAFS analysis of gold (Au) 4f was used to calibrate the applied X-ray energy. Step size energy in wide scan and C1s scan was 0.5 and 0.02 eV, respectively. Vacuum pressure was about 1.08 × 10^−8^ Pa and the X-ray irradiation angle was 0° to the sample surface normal. CasaXPS software (version 2.3.15, Casa software Ltd., Teignmouth, UK) was used to interpret the data.

#### 2.3.6. Cyclic Voltammetry Analysis

**Figure 1:**
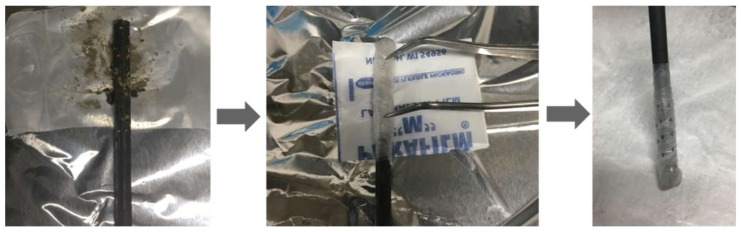
Sample preparation for cyclic voltammetric measurement: Step 1: Humin (HM) was distributed equally on a sheet of parafilm, and the graphite electrode was placed in the middle point of the sample area (about 2.5 cm of electrode’s length); Step 2: The electrode was wrapped by that sheet of parafilm, and then ~20 holes were perforated with tweezers to create at the sample area.

The measurements were performed with a potentiostat (HSV-110; Hokuto Denko, Japan) coupled with a system of three electrodes: a working graphite electrode (5 mm × 15 cm; Tokai Carbon, Japan), counter twisted platinum electrode (0.8 mm × 1 m; Nilaco, Japan), and a reference Ag/AgCl electrode (6 mm × 15 cm; Fusheng Analytical Instrument Co., Shanghai, China). The freeze-dried HM (0.025 g) was attached to the surface of the graphite electrode (2.5 cm length) by a parafilm layer. Due to the non-conductivity of parafilm, several holes were made with tweezers (Figure 1), to let the HM particles contact the electrolyte. The electrodes were then submerged in 100 mL Na_2_SO_4_ solution (0.5 M). The measurement was conducted under anaerobic conditions, with a scanning rate of 10 mV/s and potential ranging from +0.3 to −0.6 V (vs. Ag/AgCl). A background measurement was conducted with the same procedure above, with the absence of HM on the surface of the graphite electrode (blank sample).

### 2.4. Statistical Analysis

The statistical analysis to examine the significance in the correlation between the dechlorination activity and the chemical/electrochemical parameters was carried out by a Pearson’s correlation test (2-tailed) in IBM SPSS Statistics (version 21, IBM Corp., Armonk, NY, USA).

## 3. Results

### 3.1. The Influence of HM on Microbial Reductive PCP Dechlorination

**Figure 2:**
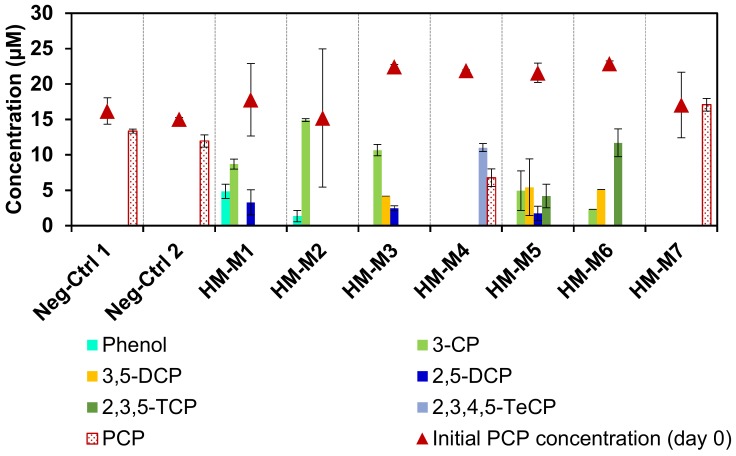
Pentachlorophenol (PCP) and metabolite concentrations on the first day (point graph) and on day 20 (column chart) of the 3rd transfer. One positive control was the culture with HM-M1, two negative controls were the culture without addition of any humin (HM) (Neg-Ctrl 1) and the culture without inoculum (Neg-Ctrl 2). No dechlorination was detected in the negative controls and the culture with HM-M7.

Figure 2 shows the influence of HM as the EEM on the microbial reductive PCP dechlorination. The culture with HM-M1 as the positive control had PCP dechlorinating activity throughout to the third generation of subcultures. There was no PCP dechlorination in the negative controls without HM and without the inoculation of the consortium. This confirmed that the anaerobic dechlorination of PCP depended on both the dechlorinating microbial consortium and HM, indicating the EEM function of HM as reported previously [8]. The different isolation methods resulted in different electron mediating abilities of the HMs, even if they were obtained from the same soil, as shown by the dechlorination rates ranging from 0.55 to 3.48 (µmol Cl^−^) L^−1^d^−1^, except for HM-M7 (Table 2). The culture with HM-M7 did not have any dechlorination activity from the first generation. Based on the PCP dechlorination rate, the order of external electron mediating ability of the seven HM conditions (from the weakest to the strongest) was as follows: HM-M7 < HM-M4 < HM-M5 = HM-M6 < HM-M2 = HM-M1 = HM-M3. The culture with HM-M4 had weak activity which caused the PCP to remain still after 20 days of incubation. The complete dechlorination metabolite, phenol, was observed only in the cultures with HM-M1 (positive control) and HM-M2.

### 3.2. Elemental Composition of HM

Table 2 shows the wide difference in the element composition of HM isolated by different methods from the same soil. The yield ranged from 1.2 to 14.5 mg-HM/g-soil. The ash content varied from 27.6% to 86.46%. The C, H, N, and O contents were in the range of 2.48–38.65%, 1.30–5.24%, 0.17–2.99%, and 8.5–25.52%, respectively. Atomic ratios also differed widely: 13.38–19.27 for C/N ratios, 1.63–6.38 for H/C ratios, and 0.50–3.29 for O/C ratios.

HM-M2 and HM-M7 had the two lowest isolation yields of 1.2 and 1.5 (mg HM/g soil), respectively, while having higher carbon contents and lower ash contents. The isolation yields of HM-M2 and HM-M7 were almost one-tenth compared to other HMs. Carbon contents in HM-M2 (38.65%) and HM-M7 (13.05%) were much higher than other HMs in this study (less than 7.0%). The low ash content in HM-M2 is due to the reaction with the strong acidic mixture, leading to a large organic fraction. The C/N ratios were in the range of 13.38 to 19.27, which agreed with a normal range of humic substances reported previously [14,33,34,35]. The correlation was not observed between the parameters based on the elemental composition and the anaerobic microbial PCP dechlorination rate.

### 3.3. FT-IR Characterization

**Figure 3:**
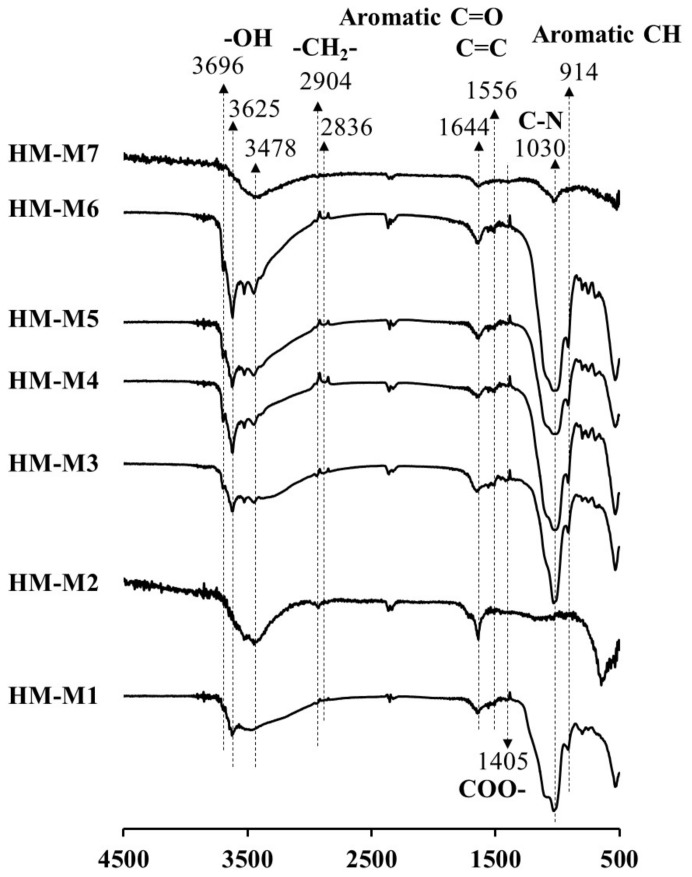
Fourier transform infrared (FT-IR) spectra of humin (HM) isolated from Kamajima paddy soil using different methods.

Figure 3 shows the FT-IR spectra of HM isolated by different methods from the same soil. The peaks at 3478, 3625 and 3969 cm^−1^ have been assigned for OH vibrations of carboxylic and alcoholic groups [36]. The peaks at 2836 and 2904 cm^−1^ have been assigned to C-H stretching motions of aliphatic groups, the peaks at 1644 and 1556 cm^−1^ to the C=O and C=C groups [37,38,39,40], and the peak at 1405 cm^−1^ to the COO^-^ group. Commonly observed were the peaks 1030 cm^−1^ (shoulder) and 914 cm^−1^, assigned as the vibration of C–N stretching and the aromatic C–H deformation, respectively [14,40]. In HM-M1, HM-M2, and HM-M7, the peak at 3696 cm^−1^ was not found. The peak at 3625 cm^−1^ also did not appear in the spectra of HM-M2 and HM-M7. Comparing to other HMs, the peak at 1030 cm^−1^ (C–N group) was weakest in the HM-M2 sample. The peak of the aliphatic group (2904 and 2836 cm^−1^) was weak (negligible) in HM-M7. The COO– group appeared weakly in all HMs, especially in HM-M2 and HM-M7. The C=O and C=C groups have been reported as potential redox-reaction centers of quinone structures in HM [14]. HM-M7, with no mediating function, had the lowest peak strength of these functional groups. Aromatic C–H groups (at 914 cm^−1^) were not found in the spectra of HM-M2 and HM-M7.

### 3.4. ESR Characterization

**Figure 4:**
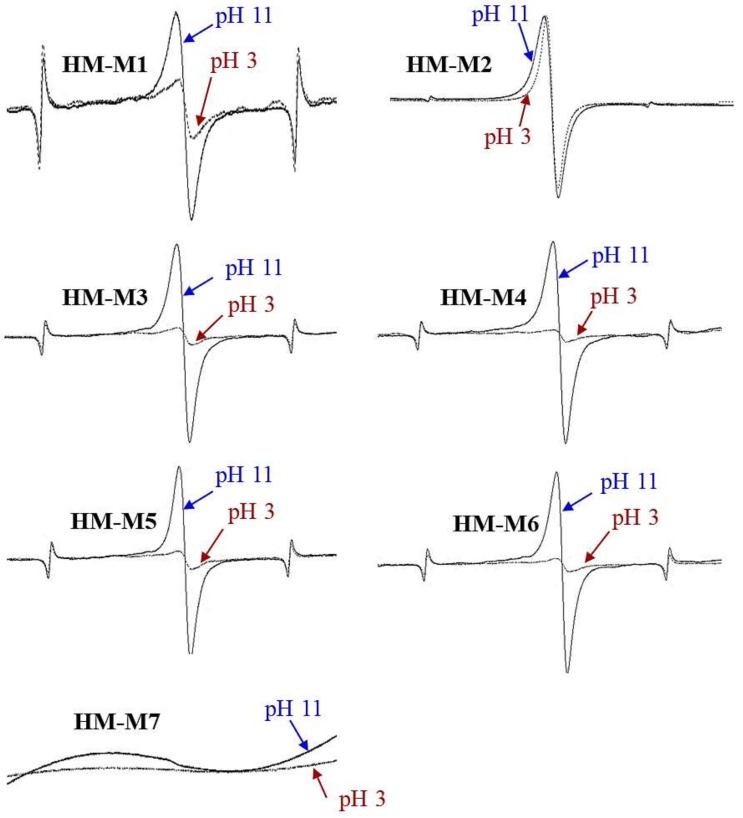
The electron spin resonance (ESR) spectra of humin (HM) isolated from the same Kamajima soil using different methods. Marker signals from the Mn calibration standard were located on two sides of organic radical peaks. No peaks were detected in HM-M7.

Figure 4 shows the ESR spectra of HM isolated from the same soil using different methods. The ESR spectra showed the signals at g = 2.00x, in the range of the g-value for organic radicals, from g = 1.99–2.01 [41], except for HM-M7. The organic radicals were observed previously in the samples of HM isolated from different soils using method 1 [14]. Signals with g-values of organic radicals were larger at pH 11 and smaller at pH 3 in most of HM samples. This result indicated the presence of quinone-type radicals. Scott et al. [42] and Paul et al. [43] found quinone moieties in the structure of humic substances. This quinone structure was suggested to be a redox-reaction center in HM, which plays a key role in the HM electron mediating function [14]. HM-M2 showed the same signal strength at pH 11 and pH 3, which indicated the presence of other types of organic radicals (other than the quinone-type radical). This type of radicals would not depend on the pH of the sample.

No signal in HM-M7 and the disappearance of marker signals might be due to the high amount of transition metals, as a result of the metal dissolution ability of DMSO/H^+^. Transition metal complex structures would cause the range of the g-signal to expand from 1.4–3.0, instead of 1.99–2.01 as is the usual range for organic radicals [41]. High concentrations of iron and cobalt were detected in the Kamajima paddy HM as reported by Zhang et al. [14]. HM-M7 did not show the signal in the range of organic radicals, neither at pH 11 nor at pH 3, suggesting HM-M7 did not contain the phenolic structure.

### 3.5. ^13^C CP/MAS NMR Characterization

**Figure 5:**
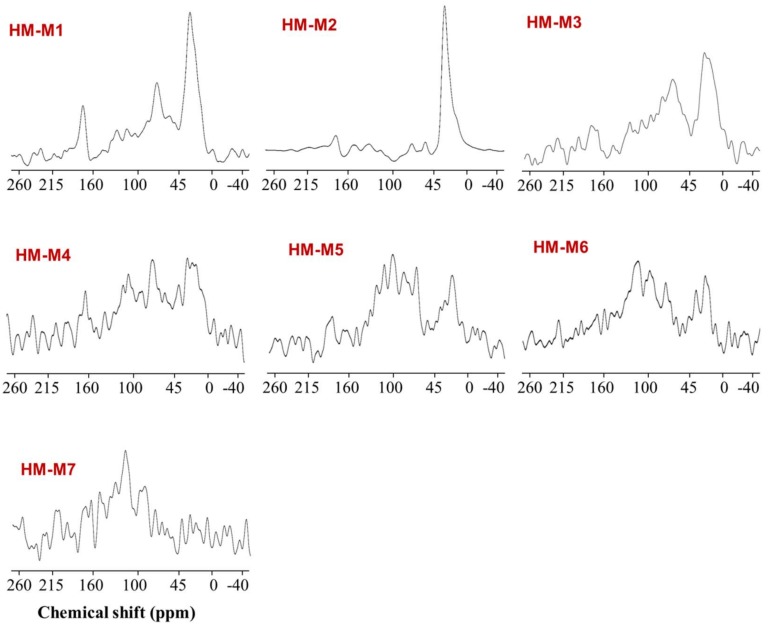
Solid-state ^13^C CP/MAS nuclear magnetic resonance (NMR) spectra of the seven HMs.

Figure 5 shows the ^13^C CP/MAS NMR spectra of the seven HMs, with Table 3 providing the information regarding the relative abundance of Carbon (%) in each HM. The spectra were divided into four functional groups, which are: aliphatic (0–45 ppm), carbohydrate (45–100 ppm), aromatic (100–160 ppm), and carboxylic carbon (160–215 ppm) [8]. For six HM, except for HM-M7, the predominant carbon binding state was an aliphatic hydrocarbon chain, occupying 34.5–68.6% of the total carbon. The relative abundance of the aromatic carbon group varied from 4.5% (HM-M3) to 20.1% (HM-M6). Largely deviated from the others, the carbon binding state of HM-M7, with no EEM function, was dominated by aromatic carbon (52.8%), but also occupied by aliphatic carbon (27.3%), and a very low proportion of carbohydrate (1.0%).

### 3.6. Characterization by Wide and Narrow Scans XPS Spectra

**Figure 6:**
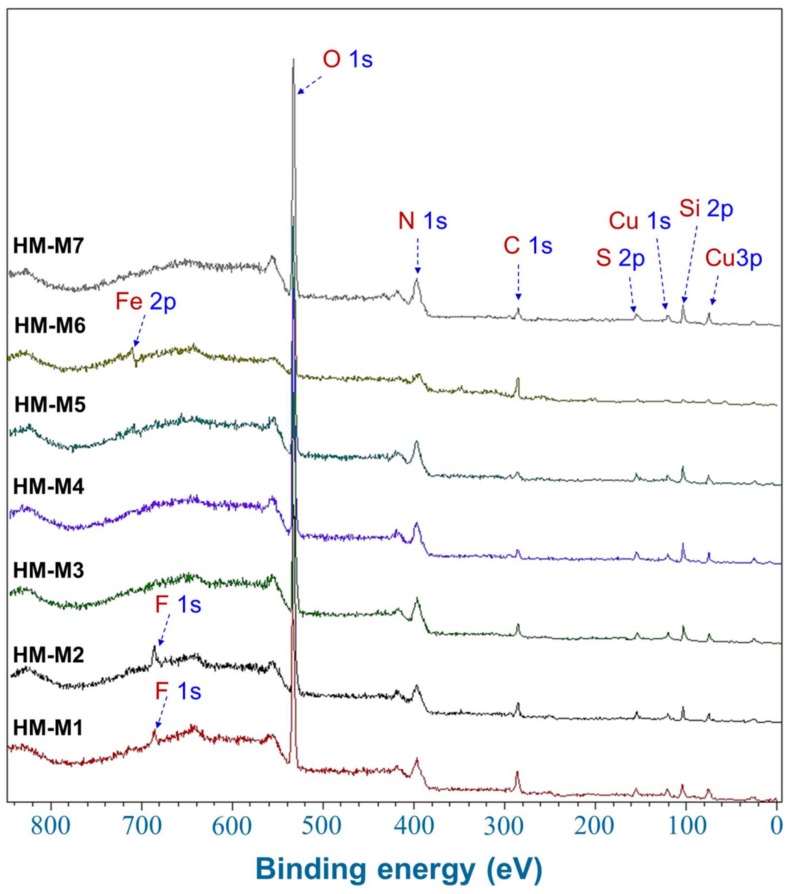
Wide scan X-ray photoelectron spectroscopic (XPS) spectra of the seven humins (HMs).

Figure 6 shows the wide scan XPS spectra of HM examined. In addition to Cu used as a matrix for the measurement, C, N, Si, S, and O were commonly observed elements in the HM, assigned according to NIST database [44]: 16.6–31.6 eV for O 2s, 74.6–79.8 eV for Cu 3p, 121.3–127.0 eV for Cu 3s, 149.3–155.3 eV for Si 2s, 160.6–172.0 eV for S 2p, 280.7–296.7 eV for C 1s, 526.7–539.8 eV for O 1s, and 682.4–694.5 eV for F 1s, ~710.9 eV for Fe 2p. F 1s was only detected in HM-M1 and HM-M2, in which HF was used in the isolation process. It should be noticed that the calculated O-content in elemental composition (Table 2) was the sum of both O and S-content in all HM, plus F-content in HM-M1 and HM-M2.

**Figure 7:**
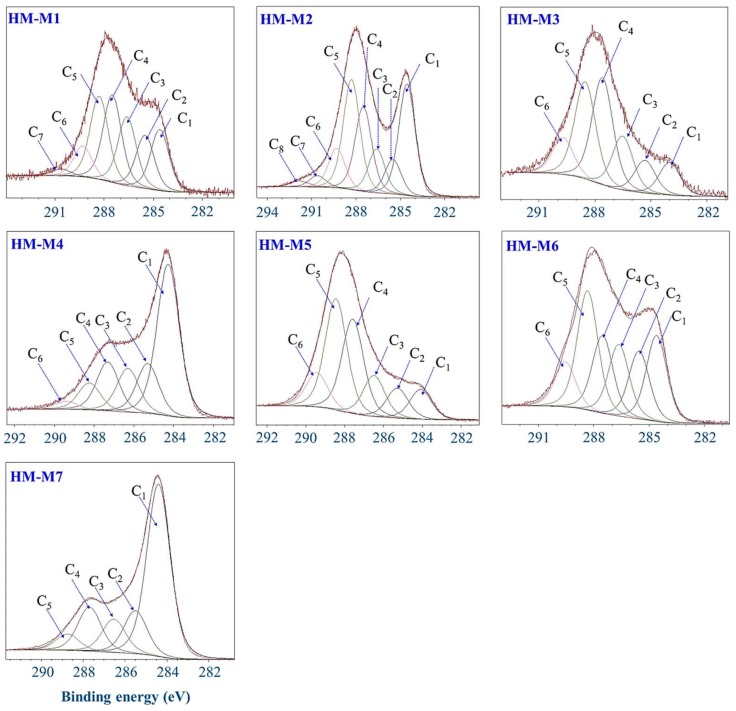
Deconvolution of C1s X-ray photoelectron spectroscopy (XPS) spectra of the seven humins (HMs).

Figure 7 shows the narrow C 1s spectra of HM obtained from the higher energy resolution scan and the deconvoluted peaks. The results of the deconvolution of peaks are summarized in Table 4. In all HM, C=C, C–C/C–H, C–O, C=O, O=C–N, O=C–O peaks were assigned. HM-M7 and HM-M4 contained highest percentage of C=C double bonds and lowest C=O, O=C–N, O=C–O in their structures. As F 1s was detected in both HM-M1 and HM-M2, CFx was assigned in C 1s XPS spectra of these two HM. HM-M7, with no electron mediating function, had the lowest proportions of C=O, O=C–N compared to other HM, and no O=C–O, which agreed with the results of FT-IR. HM-M4 was the second lowest in these functional groups in the C 1s spectrum. Both HM-M4 and HM-M7 had highest C=C percentage in their structure.

### 3.7. Characterization by Cyclic Voltammograms (CVs)

**Figure 8:**
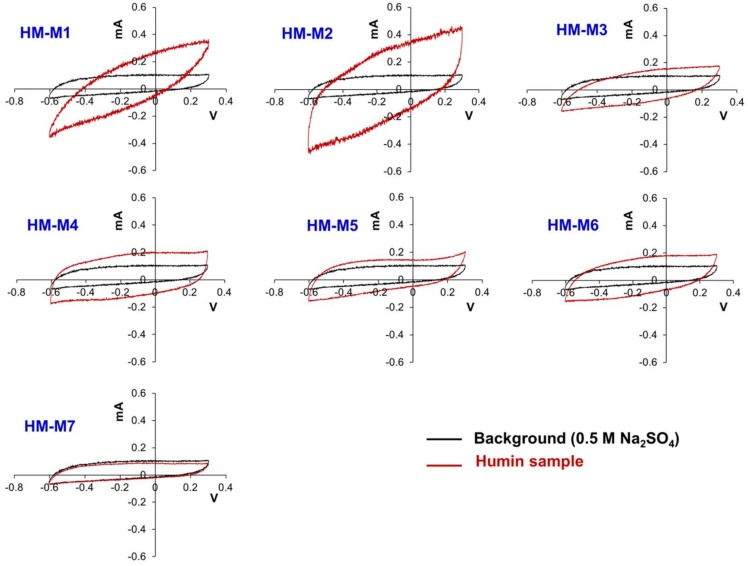
Cyclic voltammogram of the seven humin (HM) samples.

Figure 8 presents the CVs of the HMs in comparison with the background CV. All the CVs did not show any specific peak, that is, redox-active moieties were not detected in all HMs. This has been a common problem of CV measurement of HSs [51]. However, the wider area of the CV cycle represented the electron storing capacity of HMs except for HM-M7, in which the area of the CV was smaller than the background. This indicated that HM-M7 had no capacitance, which could explain why HM-M7 had no EEM function in the PCP dechlorinating culture.

## 4. Discussion

From the same soil (Kamajima paddy soil, major clay type is Kaolinite), seven HM samples were isolated with different methods. These HM samples showed the differences in the influence on the dechlorination activity of anaerobic HM-dependent PCP-to-phenol dechlorinating culture as EEMs. The results clearly indicated that the isolation methods affected the electron mediating function of the HM, and also the organic chemical structure involved in the EEM function.

HM-M7 did not possess the EEM function. The most distinctive characteristics of HM-M7 from other HMs was a lack of electric capacitance (Figure 8) despite the fact that the carbon content and organic fraction were high (Table 2). HM without electric capacitance would not function as an EEM. It has been reported that HM isolated from different soil sources (all using method 1) resulted in EEM functionality that mainly relied on the organic fraction in the HM [8]. Also, the higher organic fractions in the HM were shown to correlate with the electron mediating function [14]. However, no significant correlation was observed between the PCP dechlorination rates with the carbon or organic contents of the HMs examined here, as is found typically in the case of HM-M7, which contained an elevated HM organic fraction (Table 2) despite no EEM function. The different elemental compositions (Table 2) showed the difference in the organic chemical structure among HM samples. This result suggested that the organic chemical structure (carbon binding state), which differed depending on the isolation methods, affected the EEM function more significantly than the contents of the HM organic fraction.HM with EEM functionality commonly had electric capacitance contained C=O and C=C groups (Figure 3, FT-IR). The quinone structure exhibited evidence of semiquinone radicals (Figure 4, ESR) except for HM-M2, which was composed of the high proportions of aliphatic carbons with carbohydrate carbon and less proportions of aromatic carbon (Figure 5, Table 3, ^13^C CP/MAS NMR). HM-M2 was also predominated with carbon binding states of C=O, O=C–N, and O=C–O (Figure 7, Table 4, C1s XPS). Of these factors, the carbon binding states of O=C–N and O=C–O (C1s XPS) had significant positive correlations (*p* < 0.05) with the activity of anaerobic microbial PCP dechlorination and aromatic carbon (Figure 5, Table 3, ^13^C CP/MAS NMR), while the carbon binding state of C=C (C1s XPS) had significant negative correlations (*p* < 0.05).

The detection of quinone structures in most of HM samples suggested that the quinone structures contributed to the EEM functionality of HM, possessing electric capacitance, as reported previously [8,14]. However, a significant negative correlation between the C=O peak and C=C peak in the XPS spectra, suggested that the quinone structure was not predominant in HM. The significant negative correlation of aromatic carbon (NMR) and C=C carbon (XPS) with the activity of anaerobic microbial PCP dechlorination also suggested that a quinone structure does not mainly contribute to the electron mediating structure of HM. In addition, although HM-M2 did not contain a quinone structure (no response of organic radical signal to pH change), HM-M2 showed activity of anaerobic microbial PCP dechlorination, suggesting the other structures with EEM functionality did not have pH dependence.

The structure in the HM with EEM functionality was predominated by the aliphatic carbon with a carbohydrate carbon and partial structures of C=O, O=C–N and O=C–O, suggesting the presence of a peptidoglycan structure [52,53]. The partial structure, O=C–N and O=C–O, correlated positively with the activity of anaerobic PCP dechlorination. This suggested that the peptidoglycan structure was associated with the EEM functionality in the HM. In the basic structure of peptidoglycan, cysteine residue was considered as a candidate for the redox-active structure. Sulfur was detected in all the HM as S 2p in wide scan XPS spectra (Figure 6). Further study should be carried out to examine the sulfur association with the EEM functionality of HM.

The different isolation methods made the different parts of soils as HM samples. HM-M6 and HM-M7 were the typical example, which were isolated as different fractions using the same method from the same soil (supplemental information Figure S4), and HM-M6 possessed the EEM functionality while HM-M7 did not. The low and varying yields of HM samples ranging from 1.2 to 14.5 mg/g-soil also suggested that all the isolated HM samples here did not represent the whole solid-phase HSs (as HM). Although this incompleteness in the isolation was not avoidable during the isolation of HMs, as mentioned by Lehman and Kleber [54], it helped to explore the redox-active structure in HM for EEM functionality.

## 5. Conclusions

From the same soil, seven HM samples were isolated using different methods. These HMs were examined for EEM functionality using the anaerobic HM-dependent PCP-dechlorinating consortium. The varying rates of the PCP dechlorination suggested different external electron mediating activities of HM. HM isolated with DMSO/H+ as the soluble fraction did not have electric capacitance nor EEM functionality. Although quinone structures were considered as redox active structures in the HMs, the correlation analysis of the anaerobic microbial PCP dechlorination rate with chemical structures (carbon binding states) suggested that quinone structures did not mainly contribute to the EEM functionality of HM. The HM with EEM functionality were characterized by the predominance of aliphatic and carbohydrate carbons, with C=O, O=C–O, and O=C–N as partial structures, reflecting the structure of peptidoglycan in HM. The activity of the anaerobic microbial PCP dechlorination correlated significantly with the structures of O=C–N and O=C–O. This suggested that the EEM functionality was associated with peptidoglycan structure in HM.

## Figures and Tables

**Table 1:** Overview of isolation methods of the seven HMs.

Humin	Procedure (24 h per Wash)							Reference
	Step 1	Step 2	Step 3	Step 4	Step 5	Step 6	Step 7	
HM-M1	0.1 N NaOH	2% HF	0.1 N NaOH	2% HF	0.1 N NaOH	Distilled water	Freeze-dry	Zhang and Katayama [8]
HM-M2	Benzene/methanol (1:1 *v*/*v*)	0.1 N NaOH	HCl/HF (1:9 *v*/*v*)	0.1 N NaOH	Distilled water	Freeze-dry		Hatcher et al. [26]
HM-M3	0.1 N NaOH	Methyl isobutyl ketone	0.1 N NaOH	0.1 N NaOH	Distilled water	Freeze-dry		Rice and MacCarthy [24]
HM-M4	0.1 M H_3_PO_4_	0.1 M Na_4_P_2_O_7_	0.1 N NaOH	CHBr_3_/ethanol (1.8 g/cm^3^)	0.1 N NaOH	Distilled water	Freeze-dry	Almendros et al. [27]
HM-M5	0.1 N NaOH	Mixture of 0.1 N NaOH containing 30 g/L Na_2_SO_4_	30 g/L Na_2_SO_4_	0.1 N NaOH	Distilled water	Freeze-dry		Tsutsuki and Kuwatsuka [28]
HM-M6	0.1 N NaOH	Mixture of 0.1 N NaOH containing 6 M Urea	DMSO + H_2_SO_4_ 6% (*v*/*v*) (collected solid residue)	0.1 N NaOH	Distilled water	Freeze-dry		Hayes et al. [29]
HM-M7	0.1 N NaOH	Mixture of 0.1 N NaOH containing 6 M Urea	DMSO + H_2_SO_4_ 6% (*v*/*v*) (collected dark supernatant)	Adjust pH 9 by NaOH	0.1 N NaOH	Distilled water	Freeze-dry	Hayes et al. [29]

**Table 2:** Yield, elemental composition of humin (HM) isolated by different methods, and their influence on microbial reductive pentachlorophenol (PCP) dechlorination (generation 3, day 20).

HM	Yield (mg HM/g Soil)	Elemental Composition (%)				Ash (%)	C/N	H/C	O/C	PCP Dechlorination Rate, (µmol Cl^−^) L^−1^d^−1^
		C	H	N	O					
HM-M1	14.5	6.86	1.94	0.50	13.75	76.95	15.89	3.40	1.50	3.44
HM-M2	1.2	38.65	5.24	2.99	25.52	27.60	15.08	1.63	0.50	3.33
HM-M3	10.6	3.44	1.30	0.30	8.51	86.46	13.38	4.52	1.85	3.48
HM-M4	9.4	6.01	1.75	0.50	10.55	81.20	14.11	3.49	1.32	0.55
HM-M5	14.0	2.48	1.32	0.17	10.88	85.14	16.71	6.38	3.29	2.48
HM-M6	9.9	3.63	1.20	0.26	9.66	85.25	16.50	3.96	2.00	2.40
HM-M7	1.47	13.05	2.49	0.79	19.49	64.19	19.27	2.29	1.12	No activity

**Table 3:** Relative abundance of carbon in the seven HMs estimated from ^13^C CP/MAS NMR spectra.

	Relative Abundance of Carbon (%)			
	Aliphatic (0–45 ppm)	Carbohydrate (45–100 ppm)	Aromatic (100–160 ppm)	Carboxylic (160–215 ppm)
HM-M1	51.9	23.7	8.7	15.7
HM-M2	68.6	9.4	15.0	7.0
HM-M3	34.5	23.1	4.5	37.8
HM-M4	51.3	11.8	16.1	20.8
HM-M5	59.3	22.9	11.4	6.4
HM-M6	68.1	10.7	20.1	0.9
HM-M7	27.3	1.0	52.8	18.9

**Table 4:** Peak assignment in C1s X-ray photoelectron spectroscopy (XPS) spectra of the seven HMs.

Humin	Peak Assignment and Area Percentage (%)							
	C=C (C_1_)	C–C/C–H (C_2_)	C–O (C_3_)	C=O (C_4_)	O=C–N (C_5_)	O=C–O (C_6_)	CF_1_ (C_7_)	CF_2_ (C_8_)
HM-M1	15.6	13.6	17.6	24.4	21.2	7.8	1.7	
HM-M2	25.2	7.8	9.9	19.2	24.9	8.9	2.6	1.5
HM-M3	9.1	9.1	14.9	29.7	26.6	10.2		
HM-M4	46.2	15.2	13.3	14.8	8.2	2.4		
HM-M5	8.8	8.8	12.0	27.7	32.7	10.1		
HM-M6	18.4	14.5	15.3	16.6	25.5	9.6		
HM-M7	55.8	13.9	10.8	14.2	5.2			

Note: The assigned binding energy ranges are: 284.3 ± 0.4 eV for C=C; 285.5 ± 0.4 eV for C–C/C–H; 286.5 ± 0.4 eV for C–O; 287.5 ± 0.4 eV for C=O; 288.5 ± 0.4 eV for O=C–N; 289.5 ± 0.4 eV for O=C–O; 290.5 ± 0.4 eV for CF_1_; 291.5 ± 0.4 eV for CF_2_ [45,46,47,48,49,50]. Blank value: not assigned.

## Supplementary Materials

The following are available online at http://www.mdpi.com/1660-4601/15/12/2753/s1, Figure S1: Schematic diagram of humin isolation method 1 (HM-M1) and 2 (HM-M2), Figure S2: Schematic diagram of humin isolation method 3 (HM-M3) and 5 (HM-M5), Figure S3: Schematic diagram of humin isolation method 4 (HM-M4), Figure S4: Schematic diagram of humin isolation method 6 (HM-M6 and HM-M7).
