# Supplementary material for: Humin as an External Electron Mediator for Microbial Pentachlorophenol Dechlorination: Exploration of Redox Active Structures Influenced by Isolation Methods
Source: Int J Environ Res Public Health. 2018 Dec 5;15(12):2753. doi: 10.3390/ijerph15122753 (PMC6313380; doi:10.3390/ijerph15122753)
Supplement: Supplementary file 1 [file ijerph-15-02753-s001.pdf]

International Journal of
Environmental Research and Public Health

MDPI

Supplementary Information for

Article

Humin as an external electron mediator for microbial pentachlorophenol dechlorination: exploration of redox active structures influenced by isolation methods

Duyen Minh Pham[1,2], Arata Katayama[1,2,*]

[1] Department of Civil Engineering, Graduate School of Engineering, Nagoya University, Nagoya 464-8603, Japan
[2] Institute of Materials and Systems for Sustainability, Nagoya University, Nagoya 464-8603, Japan
* Correspondence: katayama.arata@nagoya-u.jp; Tel.: +81 52 789 5856

Number of pages: 5

Number of tables: 0

Number of figures: 4

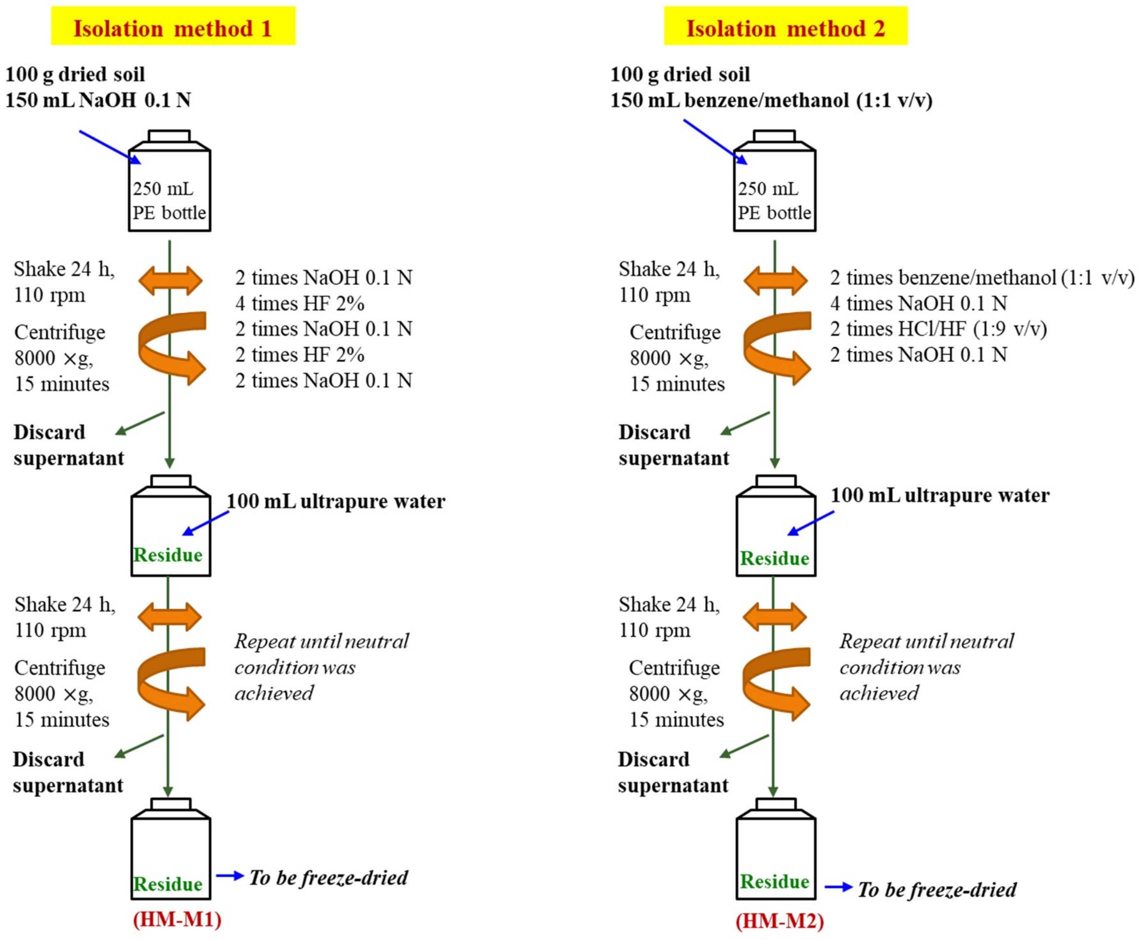

Figure S1. Schematic diagram of humin isolation method 1 (HM-M1) and 2 (HM-M2).

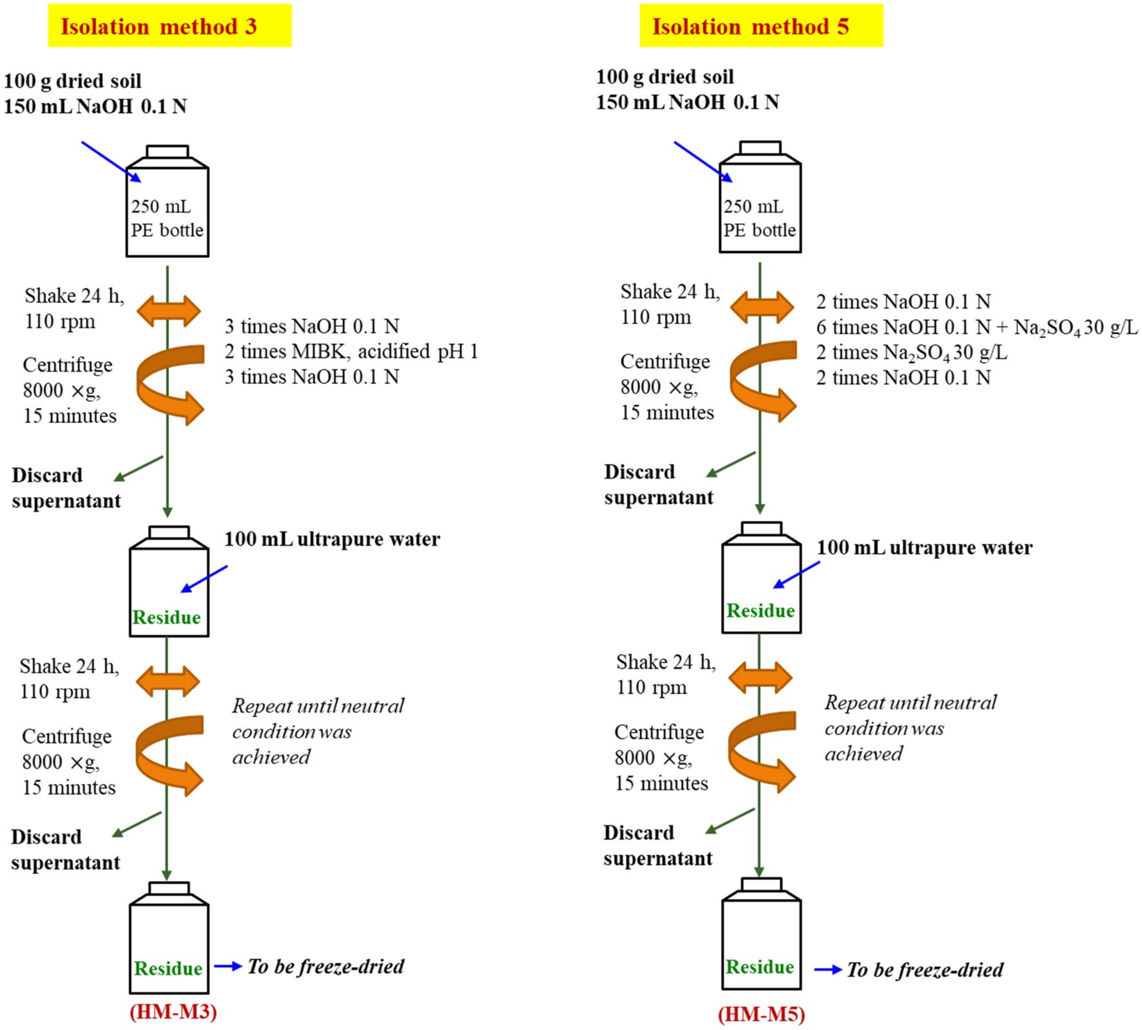

Figure S2. Schematic diagram of humin isolation method 3 (HM-M3) and 5 (HM-M5).

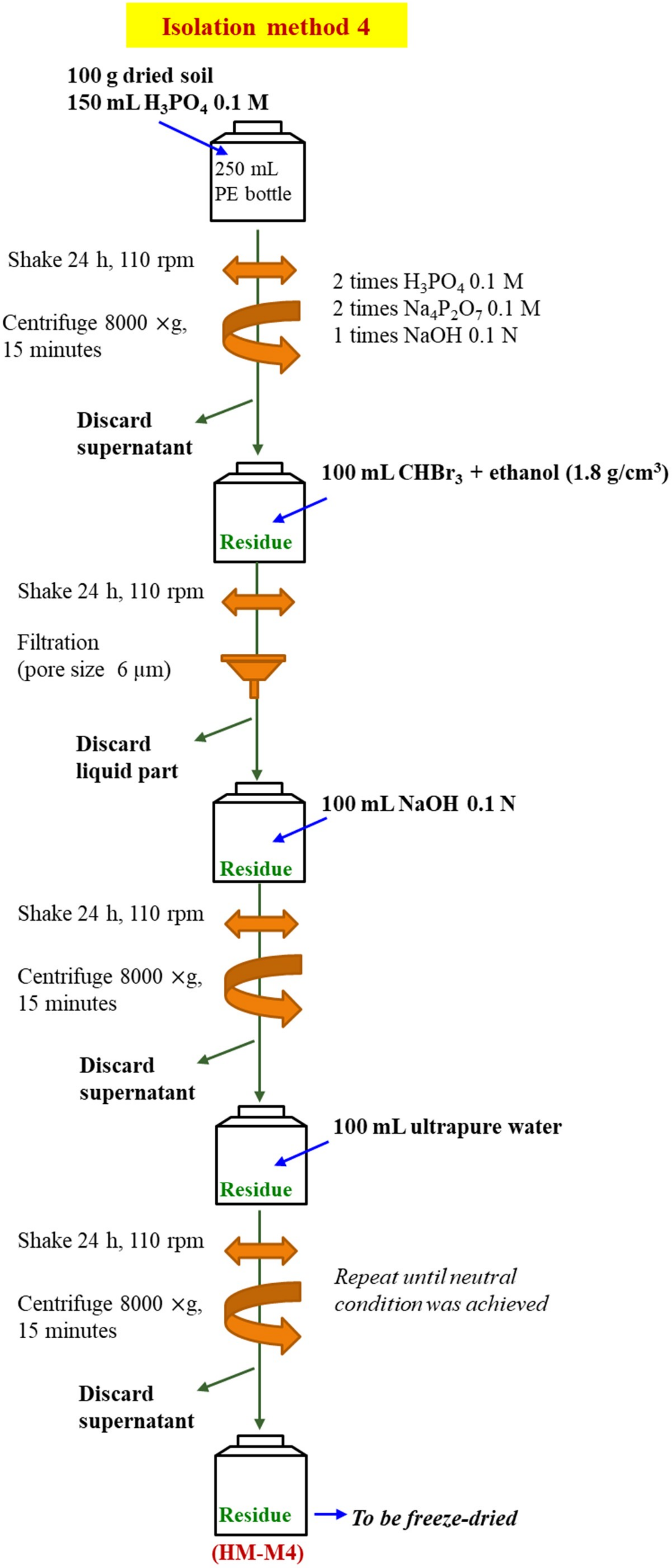

Figure S3. Schematic diagram of humin isolation method 4 (HM-M4).

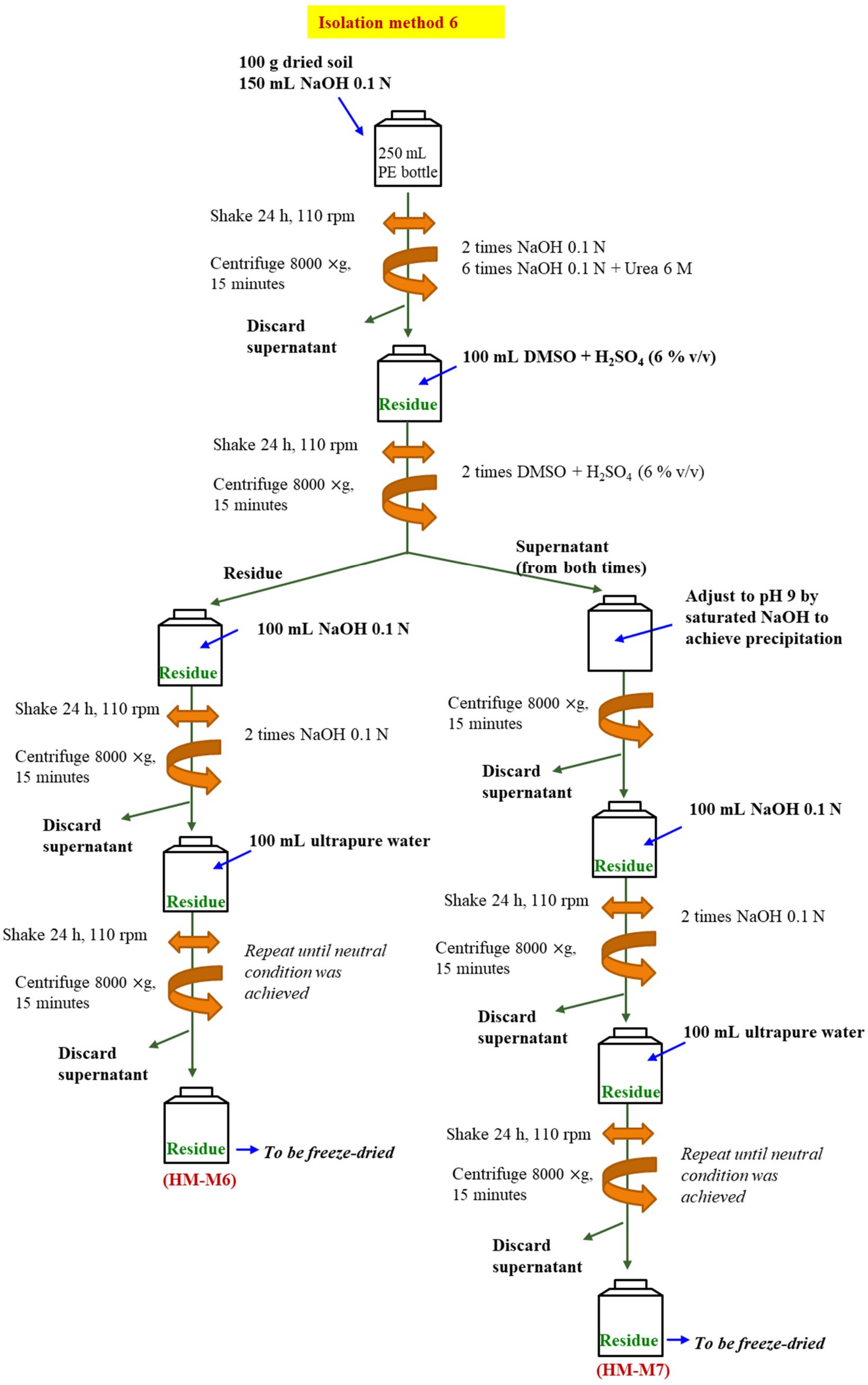

Figure S4. Schematic diagram of humin isolation method 6 (HM-M6 and HM-M7).
